# Analysis of the Gene Expression Profile of Stromal Pro-Tumor Factors in Cancer-Associated Fibroblasts from Luminal Breast Carcinomas

**DOI:** 10.3390/diagnostics10110865

**Published:** 2020-10-23

**Authors:** Noemi Eiro, Sandra Cid, María Fraile, Jorge Ruben Cabrera, Luis O. Gonzalez, Francisco J. Vizoso

**Affiliations:** 1 Unit Research, Fundación Hospital de Jove, Gijón, 33290 Asturias, Spain; sandra.cid.89@gmail.com (S.C.); maria.fraile82@gmail.com (M.F.); jorge.ruben.cabrera@gmail.com (J.R.C.); 2 Department of Pathological Anatomy, Fundación Hospital de Jove, Gijón, 33290 Asturias, Spain; a.patologica2@hospitaldejove.com

**Keywords:** cancer associated fibroblasts, tumor stroma, breast cancer, MMP11, IGF2

## Abstract

Luminal tumors are the most frequent type of breast carcinomas showing less tumor aggressiveness, although heterogeneity exists in their clinical outcomes. Cancer-associated fibroblasts (CAFs) are a key component of the tumor stroma which contribute to tumor progression. We investigated by real-time PCR the gene expression of 19 factors implicated in tumor progression. Those factors included the calcium-binding protein S100A4, several growth factors (FGF2, FGF7, HGF, PDGFA, PDGFB, TGFβ, VEGFA, and IGF2), and we also studied inflammatory cytokines (IL6 and IL8), chemokines (CCL2, CXCL12), important proteases (uPA, MMP2, MMP9 and MMP11), the nuclear factor NFκB, and the metalloprotease inhibitor TIMP1, from luminal A and luminal B breast carcinoma CAFs. We performed a similar analysis after co-culturing CAFs with MCF-7 and MDA-MB-231 breast cancer cell lines. MMP-9 and CCL2 gene expressions were higher in CAFs from luminal B tumors. We also found different patterns in the induction of pro-tumoral factors from different CAFs populations co-cultured with different cancer cell lines. Globally, CAFs from luminal B tumors showed a higher expression of pro-tumor factors compared to CAFs from luminal A tumors when co-cultured with breast cancer cell lines. Moreover, we found that CAFs from metastatic tumors had higher IGF-2 gene expression, and we detected the same after co-culture with cell lines. Our results show the variability in the capacities of CAFs from luminal breast carcinomas, which may contribute to a better biological and clinical characterization of these cancer subtypes.

## 1. Introduction

Breast cancer is a heterogeneous disease. Clinically, breast cancers are categorized into four subtypes based on their intrinsic characteristics of cancer cells to facilitate targeted therapy. Immunohistochemical determinations of estrogen receptor (ER), progesterone receptor (PR), human epidermal growth factor receptor 2 (HER2), and Ki67 status are used to categorize these tumors [1,2,3]. In accordance with the more recent criteria [4], breast cancers are classified as: (1) luminal A (ER- and/or PR-positive, HER2-negative); (2) luminal B (ER- and/or PR-positive, HER2-positive or Ki67 > 20%); (3) HER2-enriched (HER2 amplified, ER- and PR-negative); and (4) triple-negative (ER-, PR-, and HER2-negative) subtypes.

Prognosis and survival are different depending on these subtypes [3]. Luminal tumors, the most frequent (accounting for 50–60% of breast cancers) [5], are associated with the most favorable prognoses, while HER2-overexpressing and triple-negative tumors are associated with worst prognoses [6,7]. However, a biological heterogeneity exists in luminal tumors, and numerous patients with these tumors died of relapsed disease. For this reason, novel markers to improve prognosis and to decide therapeutic strategies are required.

Tumors are complex tissues composed of a heterogeneous mixture of cells, including cancer-cells and stromal cells. It is known that characteristics of stromal cells may dictate tumor outcome in breast cancer [8,9,10,11]. Cancer associated fibroblasts (CAFs) constitute the principal component of the tumor stroma. CAFs display a high proliferation rate and show capacity to facilitate tumor progression by degrading and remodeling the extracellular matrix (ECM), activating the epithelial mesenchymal transition (EMT), promoting an angiogenic shift, inducing a metabolic reprogramming toward a reverse Warburg phenotype, or stem cell trait achievement, compared with normal fibroblasts [12,13,14,15]. CAFs promote these actions by the secretion of growth factors and cytokines that influence the epithelium behavior [16,17,18,19]. In this context, CAFs can activate the NFκB signaling pathway to evoke a pro-inflammatory response, and through the secretion of IL-1β, IL-6, IL-8, and SDF-1, induce the recruitment of immune cells [20,21].

In the present study, we have investigated the gene expression of 19 factors implicated in tumor progression in CAFs from luminal breast carcinomas, and their variation after co-culture with MCF-7 and MDA-MB-231 breast cancer cell lines. Those factors were: the calcium-binding protein S100A4, several growth factors (FGF2, FGF7, HGF, PDGFA, PDGFB, TGFβ, VEGFA, and IGF2), and we also studied inflammatory cytokines (IL6 and IL8), chemokines (CCL2, CXCL12), important proteases (uPA, MMP2, MMP9, and MMP11), the nuclear factor NFκB, and the metalloprotease inhibitor TIMP1.

## 2. Material and Methods

### 2.1. Patient Selection and Their Characteristics, and Tissue Specimen Handling 

This study is a non-randomized prospective research which includes 19 women with a histological confirmed diagnosis of invasive breast carcinoma of luminal type. We selected consecutive cases of T1 or T2 invasive ductal carcinoma of luminal type with sufficient tissue for cell culture, during the period between July 2011 and August 2013, who underwent for a tumor resection as their first therapeutic approach. The exclusion criteria were: prior history of malignant tumor, excluding non-melanoma skin cancer, uterine cervix cancer in situ, ductal carcinoma in situ, or lobular carcinoma in situ of breast cancer, and a history of having received any type of therapy prior to surgery. Table 1 shows characteristics of the 19 patients with invasive breast carcinoma, all included in the analyses performed in this study and classified as luminal A (ER- and/or PR-positive, HER2-negative) or luminal B (ER- and/or PR-positive, HER2-positive or Ki67 > 20%).

During the follow-up period, a total of 6 patients developed distant metastases. The follow-up period was 43 months for patients with recurrence and 58 months for patients without. Women were treated according to the guidelines used in our Institution (Fundación Hospital de Jove). Before the evaluation of tumor samples, all patients gave their written informed consent. The study adhered to National regulations and was approved by the Fundación Hospital de Jove Ethics and Investigation Committee.

### 2.2. Cell Lines

The estrogen-independent human breast cancer cell line MDA-MB-231, and the estrogen-dependent human breast cancer cell line MCF-7, were both purchased from the American Type Culture Collection (ATCC, Rockville, MD, USA).

The MCF-7 cell line was cultured in DMEM/F-12 (Lonza, Visp, Switzerland), and the MDA-MB-231 cell line was maintained in high glucose DMEM (Sigma, St. Louis, MO, USA). Both media were supplemented with 10% FBS (PAA, North Darmouth, MA, USA) and the antibiotic mixture 1% penicillin-streptomycin solution (Gibco, Paisley, UK).

### 2.3. Primary Cell Culture 

Tumor samples were cut into 1 mm^3^ pieces and enzymatically dissociated in DMEM/F-12 supplemented with 10% FBS, 1% penicillin-streptomycin solution, and 1 mg/mL collagenase A (Roche, Hertfordshire, UK) at 37 °C for 48 h.

**Figure 1:**
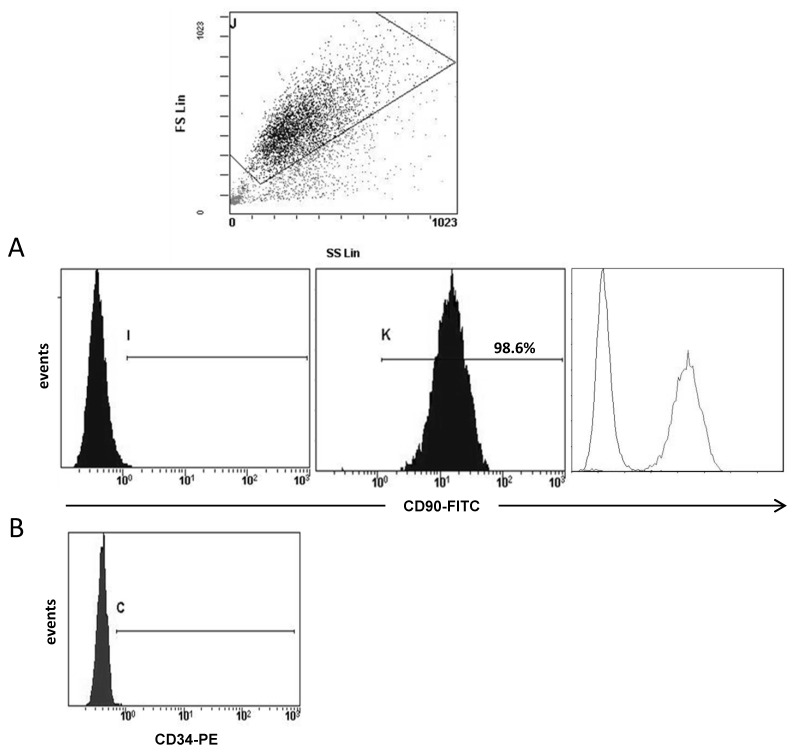
Representative example of flow-cytometry analysis of fibroblast population. At the top it shown a selection of cell population depending on the size and the cellular complexity (SS). (**A**) Negative control, positive expression for CD90 and the overlay. For this analysis all 19 patients were included. (**B**) Negative CD34 expression in a positive CD90 fibroblast population.

After enzymatic digestion, the suspension obtained was centrifuged at 400× *g* for 5 min. Cell pellet was resuspended and cultured in DMEM/F-12 supplemented with 10% FBS, 1% penicillin-streptomycin solution, 5 μg/mL insulin (Gibco), 1 μg/mL hydrocortisone (Sigma), and 5 ng/mL EGF (Invitrogen, Carlsbad, CA, USA). Cell cultures exhibited, predominantly, fibroblast morphology after three or four passages. Fibroblast culture purity was analyzed by flow-cytometry (Figure 1) using the antibody CD90 clone AS02 (Dianova, Hamburg, Germany) incubated at 1:50 (4 µg/mL) for 45 min. After, two washes with PBS 1X, cells were incubated with the secondary antibody (FITC, R & D Systems, Minneapolis, MN, USA, ref.: 0103) for 30 min. CD90 clone AS02 antibody specificity was confirmed by immunohistochemistry before being used on study samples [19]. Also, we analyzed the expression of CD34 (555822, BD Pharmingen, San Diego, CA, USA), which is expressed by hematopoietic stem cells/progenitors as well as by a multitude of other non-hematopoietic cell types, including epithelial progenitors and vascular endothelial progenitors.

### 2.4. Co-Culture Experiments

Transwell 24-well plates were used to perform co-cultures of fibroblasts with breast cancer cell lines. Fibroblast cells were plated at 2 × 10^4^ cells in the bottom of the wells, whereas breast cancer cell lines were seeded (2 × 10^4^ cells) in the 0.24 μm pore size tissue culture inserts, which were introduced into the fibroblasts-containing wells later on. Cells were co-cultured for 72 h in DMEM/F-12 supplemented with 10% FBS and 1% penicillin-streptomycin solution. Then, RNA extraction was performed using the following protocol.

### 2.5. Real Time-PCR 

“RNeasy Mini Kit” (Qiagen, Hilden, Germany) including the DNAse treatment was used according to the manufacturer’s instructions for total RNA isolation. As described previously [19], the integrity and concentration of RNA was determined spectrophotometrically by using a NanoDrop Technologies device, (Wilmington, DE, USA). First strand cDNA was synthesized using the “Transcriptor First Strand cDNA Synthesis Kit” (Roche, Mannheim, Germany) following the manufacturer’s instructions. The reverse transcription step was carried out using the following program: firstly, an incubation at 65 °C during 10 min was performed to ensure denaturation of RNA secondary structures, and then, the reverse transcription reaction was performed as follows: 10 min at 25 °C, 60 min at 50 °C, and 5 min at 85 °C.

Expressions of different factors were measured using RealTime ready custom panel plates (Roche). These custom-designed plates contained the specific primers and probes for the factors studied and for three reference genes (Table 2). The mRNA levels were measured in a LightCycler 480 II (Roche) with the following cycling conditions: 95 °C for 10 min, 45 cycles of 95 °C for 10 s, 60 °C for 30 s, and 72 °C for 1 s.

The expression was quantified using advanced relative quantification. In this method, the crossing point (Cp) is automatically calculated using the LightCycler software as the first maximum of the second derivative of the curve. A combination of two housekeeping genes (β-actin and SDHA (Succinate dehydrogenase subunit A)) as a normalization factor was used to minimize sample variability and to increase the accuracy and resolution of gene expression normalization.

### 2.6. Immunohistochemistry

To study the protein expression of IGF2, immunohistochemistry staining was performed on tissue sections of breast cancer using a TechMate TM50 autostainer (Dako, Glostrup, Denmark), a primary antibody against IGF2 (ab9574, Abcam, antigen retrieval pH6, incubation 1 h) and the EnVision Detection kit (Dako). Sections were counterstained with hematoxylin, dehydrated with ethanol and permanently coverslipped, as previously described [19].

### 2.7. Statistical Analysis

All statistical analyses were performed using PASW Statistics 18. The Kolmogorov–Smirnov Test was used to determine whether sample data were normally distributed. As data were normally distributed, comparison between groups was performed with the Student’s *t* test. Survival curves were calculated with the Kaplan–Meier method and compared by the log-rank test. Differences were considered significant when the *p*-value ≤ 0.05. The PASW Statistics program was used for all calculations.

## 3. Results

### 3.1. Molecular Profile of CAFs

**Figure 2:**
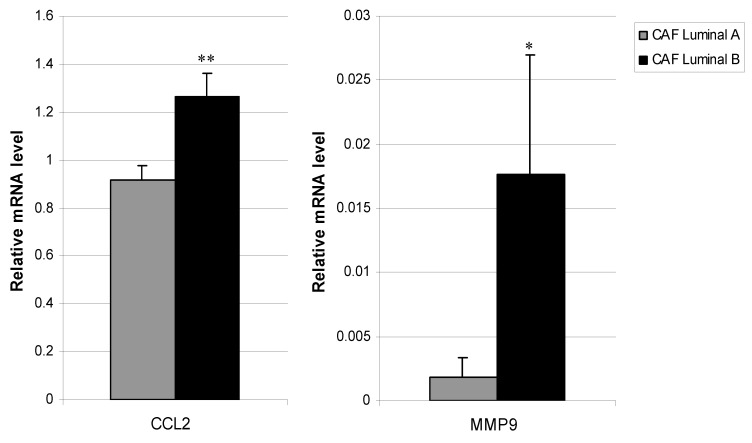
Real-time PCR analysis of factors assessed in CAFs from Luminal A and Luminal B breast cancer. Expression of CCL2 (**left**) and MMP9 (**right**). Data represent the mean ± SD (* *p* ≤ 0.05, ** *p* ≤ 0.001). For this analysis, performed in triplicate, all 19 patients were included.

We determined whether CAFs from luminal tumors presented phenotypic variability at their basal expression of several genes implicated in tumor progression (S100A4, FGF2, FGF7, HGF, PDGFA, PDGFB, TGFβ, VEGFA, IGF2, IL6, IL8, CCL2, CXCL12, uPA, MMP2, MMP9, MMP11, NFκB, and TIMP1). CCL2 and MMP9 gene expressions were significantly higher in CAFs from luminal B tumors compared to CAFs from luminal A tumors (*p* = 0.003 and *p* = 0.02, respectively, Figure 2). However, we found no significant differences in other pro-tumoral factors between CAFs from these two tumor subtypes.

### 3.2. Molecular Profile of CAFs Co-Culture with MCF-7 and MDA-MB-231 Cancer Cell Lines

**Figure 3:**
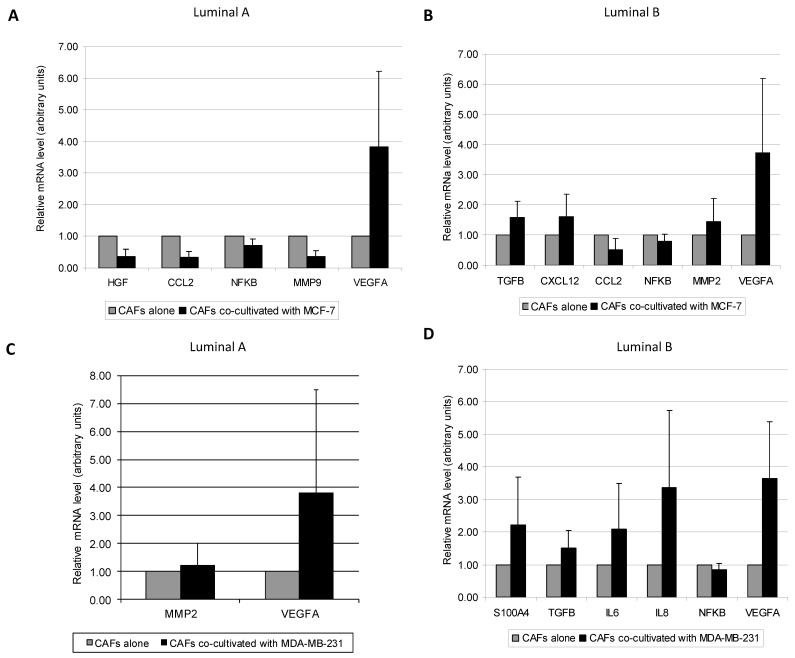
Real-time PCR analysis of factors assessed between: (**A**) CAFs from Luminal A tumors cultured alone or co-cultured with MCF-7, (**B**) CAFs from Luminal B tumors cultured alone or co-cultured with MCF-7, (**C**) CAFs from Luminal A tumors cultured alone or co-cultured with MDA-MB-231, (**D**) CAFs from Luminal B tumors cultured alone or co-cultured with MDA-MB-231. Data represent the mean ± SD. Only factors with significant differential expression are represented. *p* values are indicated in the text. Values are normalized to CAFs alone. For this analysis, performed in triplicate, all 19 patients were included.

We next examined the effect of breast tumor cell lines over different CAFs from luminal A or luminal B subtypes. We found different patterns in the expression of pro-tumoral factors depending on the CAF’s luminal type co-cultured with the different cancer cell lines. CAFs from luminal A tumors co-cultured with the luminal cell line MCF-7, showed an increase in VEGFA levels (*p* = 0.028) (Figure 3A), but a decrease in the gene expression of HGF (*p* = 0.028), CCL2 (*p* = 0.028), NFκB (*p* = 0.046), and MMP-9 (*p* = 0.028) (Figure 3A). CAFs from luminal B tumors co-cultured with MCF-7 showed a decrease in CCL2 (*p* = 0.003) and NFκB (*p* = 0.009) gene expression, while they showed an increased gene expression of other important pro-tumoral factors such as TGFβ (*p* = 0.007), CXCL12 (*p* = 0.013), MMP-2 (*p* = 0.039) and VEGF A (*p* = 0.003) (Figure 3B). When CAFs were co-cultured with the triple negative MDA-MB-231 cell line, CAFs from luminal A tumors showed an increased gene expression of MMP-2 and VEGFA (*p* = 0.028, for both) (Figure 3C), whereas CAFs from luminal B tumors showed an increased expression in VEGFA (*p* = 0.003), S100A4 (*p* = 0.019), TGFβ (*p* = 0.004), IL6 (*p* = 0.049), and IL8 (*p* = 0.009), but a decrease of NFκB expression (*p* = 0.028) (Figure 3D). All together, these data suggest that CAFs from luminal B tumors showed, globally, a higher gene expression profile of stromal pro-tumoral factors when co-cultured with any one of these two breast cancer cell lines.

### 3.3. Relationship between CAFs’ Molecular Profile and Development of Distant Metastasis

**Figure 4:**
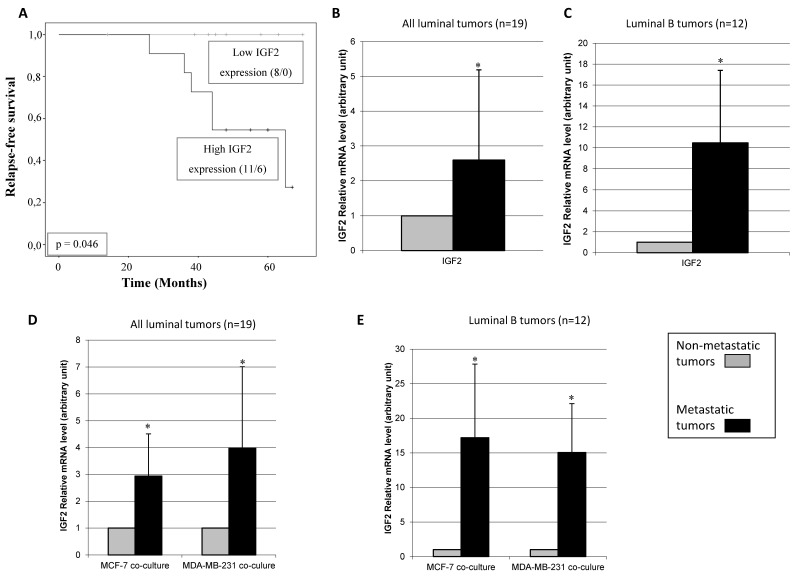
Prognostic significance of IGF2 expression studied in 19 patients with luminal breast carcinomas. (**A**) Kaplan-Meier survival curves for relapse-free survival as a function of IGF2 expression. The median value of IGF2 was taken as cut-off value. The ratio of number of events/total cases is indicated within parentheses. (**B**) Real-time PCR analysis of IGF2 assessed in CAFs from all of 19 luminal tumors (13 non-metastatic tumors and 6 metastatic tumors). (**C**) Real-time PCR analysis of IGF2 assessed in CAFs from 12 luminal B tumors (8 non-metastatic tumors and 4 metastatic tumors) * *p* = 0.08. (**D**) Real-time PCR analysis of IGF2 assessed in CAFs from all of 19 luminal tumors after co-culture with MCF-7 or MDA-MB-231. (**E**) Real-time PCR analysis of IGF2 assessed in CAFs from 12 luminal B after co-culture with MCF-7 or MDA-MB-231. * *p* < 0.05. For this analysis, performed in triplicate, all 19 patients were included.

We also compared the relapse-free survival period with the basal molecular profiling of CAFs. Six patients developed distant metastasis during the follow-up period: two patients with luminal A tumors and four with luminal B tumors. Then, we re-analyzed the gene expression of CAFs comparing those patients developing metastasis with the rest of patients. Interestingly, we found that high IGF2 gene expression (>median) in CAFs was significantly associated with a shortened relapse-free survival (Figure 4A). In addition, higher basal IGF2 gene expression was found in CAFs from metastasic tumors than in CAFs from non-metastasic tumors in all luminal tumors (Figure 4B), and especially, in luminal B tumors (Figure 4C). No significant data was obtained for luminal A tumors due to the low recurrence rate in this population and the small sample size. Finally, we also observed that higher IGF2 gene expression in CAFs from metastatic tumors remained increased after co-culture with MCF-7 or MDA-MB-231 (Figure 4D,E).

**Figure 5:**
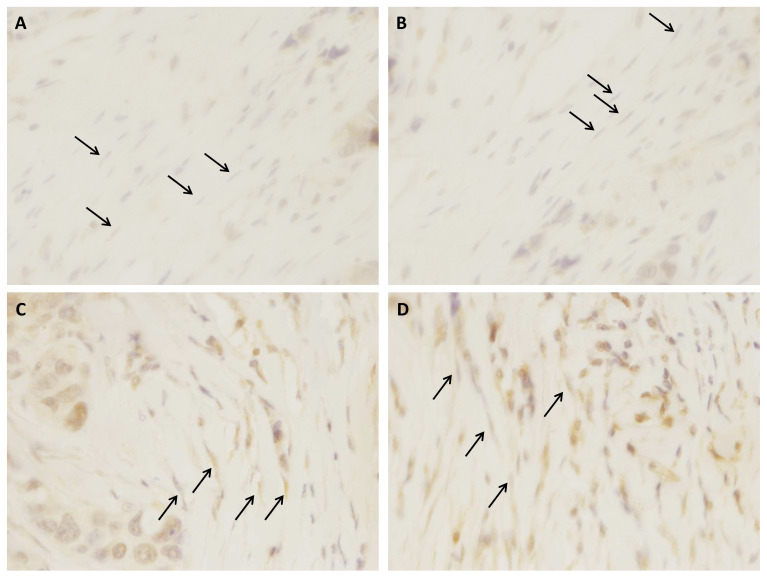
Representative examples of IGF2 (**A**,**B**) negative and (**C**,**D**) positive immunostaining in fibroblasts, corresponding to (**A**,**B**) a non-metastatic and (**C**,**D**) a metastatic luminal B breast cancer, respectively (×400). Black arrows represent fibroblasts.

In order to confirm the protein expression of IGF2, we stained primary tumors tissue sections and observed the expression by fibroblast (Figure 5). However, we could not analyze all samples (no access to tissue block for all patients) and therefore we could not establish its concordance to metastasis development.

## 4. Discussion

Today, breast tumors are categorized based on cancer cell characteristics for their management. Four main intrinsic molecular subtypes of breast cancer (luminal A, luminal B, HER2−enriched and basal-like) have been established over the last 15 years. Each of these subtypes has different features, clinical behaviors, and treatment response profiles [22]. Luminal tumors, the most frequent type (accounting for 50–60% of breast cancers), are associated with the most favorable prognoses [6,7]. However, a biological heterogeneity exists in luminal tumors, and numerous patients with these tumors died of relapsed disease. Our results indicate that CAFs from luminal B tumors show more pro-tumor characteristics compared with CAFs from luminal A tumors, which provide evidence of a possible contribution of the stroma to the different biological and clinical behavior between these tumors. Luminal A breast cancers are associated with a most favorable short-term prognosis due to its positive response to endocrine therapy [23]. Luminal B tumors are recognized to have a more aggressive clinical behavior and unfavorable prognosis compared with luminal A tumors [24,25]. In fact, many of the luminal B tumors are ER^+^/HER2^−^/high Ki-67. However, expression profiles also classify the ER^+^/HER2^+^ tumors as luminal B, and these patients receive a different therapy regimen (that incorporates targeted anti-HER2 therapy) compared to other luminal B breast cancer [26]. In addition, according to the 2013 St. Gallen Consensus, the diagnosis of a portion of patients with the luminal A subtype with poor prognosis was changed to the luminal B subtype, which was determined based on ER positivity, HER2 negativity, Ki67 expression > 14%, and PgR expression < 20% [4,27]. Thus, luminal B breast tumors (that accounted for nearly 40% of all breast cancers [28]) constitute the most heterogeneous molecular subtype at the clinical and molecular levels. Unfortunately, the immunohistochemistry of the luminal B subtype, based on intrinsic genetics characteristics from cancer cells, remains poorly defined, and it has now become a paramount importance to find new markers capable of segregating luminal tumors into clinically meaningful subgroups that may be used clinically to guide patient management. With this in mind, in the present study, we have tried to identify CAFs populations differing in their pro-tumor capabilities in these luminal tumors.

In the present study, we found variability in the gene expression of factors implicated in tumor progression in CAFs from luminal breast carcinomas (Table 1), and after co-culture with MCF-7 and MDA-MB-231 breast cancer cell lines, depending whether those CAFs are from luminal A or B tumors. CAFs from luminal B tumors showed higher gene expression of MMP-9 and CCL2 compared to CAFs from luminal A tumors. These findings are relevant if we consider that MMP-9 (also known as Gelatinase B) is related to tumor invasion and metastasis by its capacity to degrade the type IV collagen found in basement membranes [29], and to induce angiogenesis [30]. Likewise, MMP-9 has been associated with tumor aggressiveness and/or poor prognostic in patients with breast cancer [31,32,33]. On the other hand, CCL2 (monocyte chemoattractant protein 1, MCP1) is a chemokine that exerts a potent chemotactic, stimulatory, and mitogenic effects on mononuclear cells [34]. High levels of CCL2 in the tumor microenvironment [35], as well as high circulating concentrations of this chemokine, have been associated with poor prognosis in breast carcinoma patients [36]. CCL2 also stimulates the migration of mammary carcinoma cell lines [37], and mediates the recruitment of specific monocyte populations that support the establishment of metastatic disease [38].

Especially relevant were our findings showing that different CAFs populations differentially expressed pro-tumor factors depending on co-cultured cancer cell lines. Thus, after co-culture with the luminal cell line MCF-7, CAFs from luminal A tumors showed an increase in VEGFA levels, but a decreased expression levels of other pro-tumor genes (HGF, CCL2, NFκB, and MMP-9) while CAFs from luminal B tumors although showed decreased levels of CCL2 and NFκB gene expression, but increased gene expression of other important pro-tumor factors like TGFβ, CXCXL12, MMP-2, and VEGF A. Likewise, different patterns of gene expression were found in CAFs from Luminal A tumors, with increased expression of MMP-2 and VEGFA, or CAFs from luminal B tumors, with increased levels of VEGFA, S100A4, TGFβ, IL6, and IL8, but decreased levels of NFκB, after being co-cultured with the cell line MDA-MB-321.

In general, CAFs from luminal B tumors showed higher expression of pro-tumor factors when co-cultured with breast cancer cell lines, which is in accordance with the fact that these tumors are associated with higher tumor aggressiveness. These overexpressed factors are of importance promoting several biological aspects of tumor progression. TGFβ is a well-known cytokine that induces malignant mammary epithelial cell to undergo EMT, leading to the acquisition of highly migratory, invasive and metastatic phenotypes [39]. It has been also described that TGFβ can induce the expression of different proteins, such as growth factors, cytokines, and ECM proteins in CAFs, which promote tumor development in the adjacent epithelium [40]. CXCL12 is a highly pleiotropic chemokine, influencing a variety of biological processes through the interaction with its receptors CXCR4 and CXCR7. CXCL12–CXCR4 signaling has been shown to play a role in tumor growth, invasion, angiogenesis and bone marrow cell recruitment [41,42,43,44]. In line with this, it has been demonstrated that co-implantation of tumor cells with fibroblasts expressing CXCL12 enhances tumor growth [41] and CXCL12 overexpression has been linked to increased metastasis and poor prognosis [45]. In addition, a number of different agents have been used to block the CXCL12–CXCR4 interaction to inhibit metastasis [46,47,48].

MMP-2 (Gelatinase A) together with MMP-9 (Gelatinase-B) are related to tumor invasion and metastasis by their special capacity to degrade type IV collagen in basement membrane [29], and to induce angiogenesis [30]. MMP-2 production increased during the early phase of breast cancer [49], but also high levels of MMP-2 have been related to poor outcome in breast carcinomas [50]. VEGFA is a potent regulator of angiogenesis and thereby involved in the development and progression of solid tumors, such as breast cancer [51,52,53]. S100A4, a member of the S100 calcium-binding protein superfamily, has been described as a key player in promoting metastasis, acting as an angiogenic factor, inducing cell motility, and increasing the expression of MMPs [54,55,56]. In fact, elevated levels of the calcium-binding protein S100A4 have been associated with poor patient survival in breast cancer patients and to induce metastasis in rodent models [57,58].

Interestingly, we have observed high expression levels of IL6 and IL8 in CAFs from luminal B tumors after co-culture with MDA-MB-231 cells. Previous reports showed that both cytokines are important to maintain the aggressiveness of MDA-MB-231 cells, a highly tumorigenic cell line which appears to be devoid of stemness-related features [59]. In addition, clinical studies have showed that both high levels of IL-6 or IL-8 have been associated with breast cancer recurrence [60,61,62,63]. In addition, several strategies to disrupt IL-6 signaling [64] or IL-8 signaling [65], in vitro or in vivo, significantly reduce tumor growth and metastasis in breast cancer cells.

Together, these gene expression data may to contribute to a better characterization of tumor stroma of luminal breast carcinomas.

Finally, we reclassified the CAFs gene expression analysis considering patients developing or not distant metastasis during the follow-up period. An obvious limitation of the present study is the reduced number of patients who developed distant metastasis during the follow-up period. Nevertheless, and interestingly enough, we have observed that CAFs from metastatic luminal tumors had the highest IGF-2 gene expression, either in basal levels or after being co-cultured with MCF-7 or MDA-MB-231 cell lines. These findings seem to be in agreement with previous data revealing that IGF2, which is overexpressed in a wide spectrum of human cancers, is associated with more aggressive tumors [66,67] and with a poor prognosis [68]. IGF-2 promotes excessive growth and anti-apoptotic effects in cancer cells [69]. In fact, it has been shown that IGF-2 signaling is important to stimulate the proliferation of MCF-7 [70,71] and MDA-MB-231 [72]. In addition, IGF-2 has been recently implicated in a positive feedback circuit as a critical mechanism to increase stemness and maintain breast cancer cells [73]. More importantly, it has been suggested that CAFs derived from breast metastatic tumors have increased protumorogenic properties due to the increased expression of IGF2 [74]. On the other hand, there are several lines of evidence linking IGF signaling and luminal tumors. In one large follow-up study on breast cancer in postmenopausal women, serum levels of IGF-2, but not IGF-1, were positively associated with oestrogen receptor-positive breast cancer risk [75]. Likewise, it has been reported that both the co-expression [76] and interactions [77] of IGF1R (receptor for IGF-1 and IGF-2) and ER signaling systems suggest a role for the IGF1R network in the resistance to endocrine therapy [76,78]. It is also remarkable that IGF-2 is known to play an important role during fetal growth and development, and its expression in malignant tumors might implicate a more primitive cellular phenotype [79]. Taken together, our results suggest that IGF-2 expression may be a potential useful marker of tumor aggressiveness and poor prognosis in luminal tumors.

In summary, our data may contribute to a better biological characterization of the interaction between cancer cells and tumor stroma in luminal breast carcinomas. In this way, our results allow us to consider that, among the complex array of soluble factors produced by CAFs, it could arise possible new prognostic markers and therapeutic targets for luminal breast cancer.

## Figures and Tables

**Table 1:** Clinicopathological characteristics of the 20 patients with invasive breast carcinoma.

Characteristics	Luminal A	Luminal B	
	Nº (%)	Nº (%)	*p* Value
All patients	7	12	
Median age (years)			0.515
<62	4 (57.1%)	5 (41.7%)	
>62	3 (42.9%)	7 (58.3%)	
Tumor size			
T1	3 (42.9%)	1 (8.3%)	
T2	4 (57.1%)	10 (83.4%)	
T3	0 (0.0%)	1 (8.3%)	
Histological grade			0.082
Well differentiated (I)	2 (28.6%)	0 (0%)	
Moderately differentiated (II)	4 (57.1%)	6 (50.0%)	
Poorly differentiated (III)	1 (14.3%)	6 (50.0%)	
Nodal status			0.960
Negative	3 (42.9%)	5 (41.7%)	
Positive	4 (57.1%)	7 (58.3%)	
Estrogen receptors			-
Negative	0 (0%)	0 (0%)	
Positive	7 (100%)	12 (100%)	
Progesterone receptors			0.253
Negative	0 (0%)	2 (16.7%)	
Positive	7 (100%)	10 (83.3%)	
HER2			0.149
Negative	7 (100%)	9 (75.0%)	
Positive	0 (0%)	3 (25.0%)	
Ki67			0.020
<20%	4 (57.1%)	1 (8.3%)	
≥20%	3 (42.9%)	11 (91.7%)	

Chi-square test applied.

**Table 2:** Factors analyzed and main roles.

Gene Symbol	References	Gene Name	Main Role
S100A4	110779	S100 calcium binding protein A4	Invasion
TGFβ	101210	Transforming growth factor beta	Inflammation
HGF	108357	Hepatocyte growth factor	Cell growth/Invasion
FGF2	118274	Fibroblast growth factor 2 (basic)	Angiogenesis
FGF7	113109	Fibroblast growth factor 7	Cell growth/Invasion
PDGFA	110648	Platelet-derived growth factor alpha	Angiogenesis
PDGFB	110713	Platelet-derived growth factor beta	Angiogenesis
uPA	109571	Urokinase-type plasminogen activator	ECM remodelling
IL6	113614	Interleukin 6	Inflammation
IL8	103136	Interleukin 8	Inflammation
CXCL12	110618	Chemokine (C-X-C motif) ligand 12	Inflammation
CCL2	141156	Chemokine (C-C motif) ligand 2	Inflammation
NFkB	100646	Nuclear factor kappa B	Inflammation/Tumor growth
MMP2	103899	Matrix metalloproteases 2	ECM remodelling
MMP9	139820	Matrix metalloproteases 9	ECM remodelling
MMP11	103163	Matrix metalloproteases 11	ECM remodelling
TIMP1	103847	Tissue inhibitor of metalloproteases 1	ECM remodelling
VEGFA	140392	Vascular endothelial growth factor A	Angiogenesis
IGF2	113548	Insulin-like growth factor 2	Cell growth
ACTB	101125	Actin, beta	-
SDHA	102136	Succinate dehydrogenase complex, subunit A, flavoprotein	-
